# Impact of early headache neuroimaging on time to malignant brain tumor diagnosis: A retrospective cohort study

**DOI:** 10.1371/journal.pone.0211599

**Published:** 2019-02-01

**Authors:** Matthew R. Carey, Brian C. Callaghan, Kevin A. Kerber, Lesli E. Skolarus, James F. Burke

**Affiliations:** 1 University of Michigan Medical School, University of Michigan, Ann Arbor, Michigan, United States of America; 2 Department of Neurology, University of Michigan, Ann Arbor, Michigan, United States of America; George Washington University, UNITED STATES

## Abstract

**Background:**

Neuroimaging for headaches is both common and costly. While the costs are well quantified, little is known about the benefit in terms of diagnosing pathology. Our objective was to determine the role of early neuroimaging in the identification of malignant brain tumors in individuals presenting to healthcare providers with headaches.

**Methods:**

This was a retrospective cohort study using administrative claims data (2001–2014) from a US insurer. Individuals were included if they had an outpatient visit for headaches and excluded for prior headache visits, other neurologic conditions, neuroimaging within the previous year, and cancer. The exposure was early neuroimaging, defined as neuroimaging within 30 days of the first headache visit. A propensity score-matched group that did not undergo early neuroimaging was then created. The primary outcome was frequency of malignant brain tumor diagnoses and median time to diagnosis within the first year after the incident headache visit. The secondary outcome was frequency of incidental findings.

**Results:**

22.2% of 180,623 individuals had early neuroimaging. In the following year, malignant brain tumors were found in 0.28% (0.23–0.34%) of the early neuroimaging group and 0.04% (0.02–0.06%) of the referent group (*P*<0.001). Median time to diagnosis in the early neuroimaging group was 8 (3–19) days versus 72 (39–189) days for the referent group (*P*<0.001). Likely incidental findings were discovered in 3.17% (3.00–3.34%) of the early neuroimaging group and 0.66% (0.58–0.74%) of the referent group (*P*<0.001).

**Conclusions:**

Malignant brain tumors in individuals presenting with an incident headache diagnosis are rare and early neuroimaging leads to a small reduction in the time to diagnosis.

## Introduction

Headache diagnoses are common, resulting in numerous outpatient visits, emergency department encounters, prescriptions, and diagnostic tests [1–5]. From 2007 to 2010, there were 51.1 million headache visits in the United States, resulting in 6.3 million neuroimaging studies that cost $3.9 billion [3]. However, the potential downsides of overly broad neuroimaging are numerous: unnecessary costs, inconvenience for the individuals, and identification of incidental findings that may result in downstream harms [6–8]. Therefore, the appropriateness of neuroimaging in many individuals with headaches has been called into question [9, 10]. The American Board of Internal Medicine Foundation *Choosing Wisely* campaign has identified neuroimaging for headaches as an area for more judicious use [11, 12].

While these potential risks of headache neuroimaging are well-documented, neither the potential risks nor the potential benefits of headache neuroimaging are well quantified. The most common rationale for performing headache neuroimaging is to attempt to detect a treatable cause [13]. Malignant brain tumors, such as gliomas and metastatic brain tumors, carry a poor prognosis, and survival can be modestly improved with aggressive interventions including surgery, chemotherapy, and radiation [14–18]. In contrast, other findings, such as arteriovenous malformations, should rarely, if ever, lead to changes in treatment strategy as interventional therapy does not reduce the risk of death or stroke compared to observation [19]. Still others, such as intracranial bleeding, usually present in the emergency setting. Our study focuses on the use of early outpatient neuroimaging to detect malignant brain tumors as they are the best example of findings that can be discovered using neuroimaging in a primary care setting and where diagnosis leads to a substantive shift in treatment.

The low rates of malignant brain tumors in individuals with headaches makes a randomized, controlled, clinical trial challenging. Consequently, we designed an observational study using a large claims-based dataset to explore this question. We seek to better quantify both the potential benefits of headache neuroimaging by characterizing the frequency of malignant brain tumors and the time to diagnosis, as well as the potential risks of neuroimaging by quantifying the rate of likely incidental findings.

## Methods

We performed a retrospective cohort study using de-identified administrative claims data from the Clinformatics Datamart (OptumInsight: Eden Prairie, MN), a comprehensive database for all enrollees at a large, national insurer for years 2001 to 2014 [20]. The use of a de-identified database was determined to be exempt by the University of Michigan Institutional Review Board. An online appendix provides additional methodologic information (see S1 File).

### Study population

Our study population included all individuals with incident headaches presenting in an outpatient setting who did not have any insurance claims for headache diagnoses in the prior 2 years and no history of other conditions that would merit neuroimaging. Headaches were identified using the primary diagnosis code based on the following International Classification of Diseases, 9^th^ revision, Clinical Modification (ICD-9-CM) diagnosis codes: 339.xx, 346.xx, and 784.0x. Conditions meriting neuroimaging were identified using Agency for Healthcare Research and Quality (AHRQ) Healthcare Cost and Utilization Project Clinical Classification Software groupings of ICD-9-CM diagnosis codes or individual diagnosis codes and included: multiple sclerosis; other hereditary or degenerative nervous system conditions; epilepsy/convulsions; acute cerebrovascular disease; transient cerebral ischemia; intracranial injury; and delirium, dementia, and amnestic or other cognitive disorders [21]. The population was also limited to individuals with at least 2 years of continuous data after the incident headache or a malignant brain tumor diagnosis any time after the incident headache encounter. Individuals were excluded if they had neuroimaging in the prior year, a visit to a neurologist prior to the incident headache visit, or a prior cancer diagnosis.

### Primary exposure

Our primary exposure was neuroimaging within 30 days of first headache visit. Individuals were assigned to one of two groups: those who received neuroimaging within 30 days of the headache visit and those who did not. Neuroimaging was defined as computed tomography (CT) or magnetic resonance imaging (MRI) of the head/brain using Current Procedure Terminology (CPT) and ICD-9-CM procedure codes.

### Outcomes

Our primary outcomes were frequency of and median time to diagnosis of malignant brain tumors. We included primary malignant brain tumors and metastases. An approach to using administrative data to identify tumors has been characterized by others [22]. Given the challenges associated with using claims data for identifying malignant brain tumors without imaging reports or histologic evidence, we employed three different definitions for both primary malignant and metastases with varying levels of restriction to evaluate the sensitivity of our results to the definition chosen (base and more-/less-restrictive). See Table 1 for definitions. The malignancy diagnosis date was the date of the first claim that met the definition criteria.

**Table 1:** Criteria for flagging primary brain malignancies and brain metastases using the base definition, more restrictive definition, and less restrictive definition.

	Primary brain malignancy	Brain metastasis
Base definition	- Primary malignant brain tumor diagnosis AND radiation therapy AND surgery AND no brain metastasis diagnosis; OR - Temozolomide AND no brain metastasis diagnosis	- Metastatic brain tumor diagnosis AND 1 out of 3 of the following: (1) radiation, (2) surgery, (3) chemotherapy AND no primary brain malignancy diagnosis
More restrictive	- Primary malignant brain tumor diagnosis AND radiation therapy AND surgery AND temozolomide (on or after January 1, 2008)^a^ AND no brain metastasis diagnosis; OR - Primary malignant brain tumor diagnosis AND radiation therapy AND surgery (before January 1, 2008)^a^ AND no brain metastasis diagnosis	- Metastatic brain tumor diagnosis AND 2 out of 3 of the following: (1) radiation, (2) surgery, (3) chemotherapy AND no primary brain malignancy diagnosis
Less restrictive	- Primary malignant brain tumor diagnosis AND (radiation therapy OR surgery) AND no brain metastasis diagnosis; OR - Temozolomide AND no brain metastasis diagnosis; OR - Radiation therapy AND surgery AND no brain metastasis diagnosis	- Metastatic brain tumor diagnosis

^a^ Date criteria included to account for the publication of the Stupp treatment protocol in 2005[16]

Our secondary outcome was radiographic findings that were likely incidental. Likely incidental findings included benign neoplasms (e.g., benign meningiomas and benign pituitary adenomas) and other benign intracerebral findings (e.g., arteriovenous malformations). Although it is possible that these findings would necessitate intervention, we used the term “likely incidental” as a conservative approach involving serial follow-up is often recommended in these situations [23].

All-cause mortality was an explanatory outcome. We anticipated that mortality would be higher among individuals who underwent early imaging, as unmeasured factors leading to worse outcomes would likely be more common in this group. If overall mortality were lower with early neuroimaging, but not lower among individuals diagnosed with malignant brain tumors, it would raise the possibility that neuroimaging may be influencing outcomes through a non-malignancy related pathway. To explore this possibility, survival was calculated from the date of the incident headache visit to avoid lead-time bias attributed to early imaging. A separate subset of the OptumInsight Clinformatics Datamart was used for this analysis that included the year and month of death that was not linked to the main analytical dataset for privacy reasons.

### Propensity score matching

We used propensity score matching to ensure balance on measured covariates between the early neuroimaging and referent groups [24]. Matching was performed using a 1:1 nearest-neighbor procedure without replacement after estimating a propensity score for each individual.

Propensity scores were estimated using a multi-level logistic regression with a dependent variable of whether an individual had early neuroimaging and with a random Hospital Referral Region (HRR)-level intercept to account for regional variation in neuroimaging availability and practices. The following independent variables were used: age at time of headache diagnosis, sex, 17 individual Charlson comorbidities [25], year of diagnosis, migraine versus other headache type, cancer incidence rate in the individual’s county, smoking history, and neurological symptoms prior to the incident headache visit (aphasia, voice disturbance, other speech disturbance, hallucinations, convulsions, dizziness, memory loss, altered mental status, abnormal involuntary movements, disturbances of smell/taste, abnormalities of gait, lack of coordination, transient paralysis of limb, neglect, facial weakness, disturbances of skin sensation, and generalized weakness). Cancer incidence was based on data from the National Cancer Institute Surveillance, Epidemiology, and End Results Program [24, 26].

For privacy reasons, the dataset with date of death information does not contain geographic variables and so the early neuroimaging and referent groups were matched 1:1 on all variables listed above except HRR and local cancer rates.

### Statistical analyses

Descriptive statistics prior to and after propensity matching with standardized differences were conducted to compare individual demographics and clinical characteristics between the early neuroimaging and referent groups. To compare the time to malignant brain tumor diagnosis for those with and without early neuroimaging, the Kruskal-Wallis H-Test was used. Even after propensity score matching, we assume that the early imaging group would have higher disease severity than the referent group and thus we anticipated higher mortality in the early imaging group. To explore whether overall treatment patterns in the early imaged group may lead to sufficiently improved outcomes to overcome this bias, Kaplan-Meier survival analyses were performed to compare the all-cause mortality among the early neuroimaging and referent groups. Cox proportional hazards models were used to estimate the impact of early imaging on the risk of mortality. All statistical tests used a 2-sided α of 0.05. SAS 9.4 (SAS Institute, Inc.: Cary, NC) and Stata/MP 14 (StataCorp LP: College Station, TX) were used for data management and analyses.

## Results

### Descriptive statistics

180,623 individuals with incident headaches were included in this analysis. early neuroimaging was performed in 22.2% of the study population. Neuroimaging was nearly evenly divided between MRI scans (53.7%) and CT scans (51.5%) with some individuals receiving both. Among individuals who did not undergo early neuroimaging, 18% received it at a later date. S1 Table provides descriptive statistics for the early neuroimaging and referent groups. After propensity score matching, the early-imaging and referent groups each had 40,028 individuals. Table 2 provides descriptive statistics for the matched groups.

**Table 2:** Descriptive statistics for the “Early neuroimaging” and “No early neuroimaging” groups (matched).

	Early neuroimaging (n = 40,028)	No early neuroimaging (n = 40,028)	Standardized difference
Female, %	59.94	59.44	0.010
**Enrollment duration categories**			-0.029
<6 years, %	34.12	33.14	
6–8 years, %	27.19	26.92	
8+ years, %	38.69	39.94	
**Age categories**			0.003
18–34, %	29.51	28.94	
35–49, %	38.79	39.26	
50–64, %	19.99	20.76	
65+, %	11.71	11.03	
Migraine diagnosis, %	15.15	15.47	-0.009
Prior neurological symptoms, %	12.58	13.30	-0.021
Smoking history, %	5.92	6.02	-0.004
**Charlson comorbidities**			
Myocardial infarction, %	0.58	0.53	0.007
Congestive heart failure, %	0.96	0.95	0.001
Peripheral vascular disease, %	0.02	0.02	0.002
Cerebrovascular disease, %	0.02	0.01	0.008
Dementia, %	<0.01	—	0.007
Chronic pulmonary disease, %	15.38	15.34	0.001
Rheumatic disease, %	1.57	1.47	0.008
Peptic ulcer disease, %	1.18	1.15	0.002
Mild liver disease, %	0.35	0.34	0.002
Diabetes without chronic complication, %	7.70	7.71	<0.001
Diabetes with chronic complication, %	1.25	1.22	0.002
Hemiplegia or paraplegia, %	0.04	0.02	0.001
Renal disease, %	1.48	1.43	0.004
Any malignancy, including lymphoma and leukemia, except malignant neoplasm of skin, %	0.02	0.02	0.003
Moderate or severe liver disease, %	0.07	0.06	0.005
Metastatic sold tumor, %	—	<0.01	-0.007
AIDS/HIV, %	0.29	0.26	0.005

### Malignant brain tumor diagnosis

Malignant brain tumors were identified in 0.22% of individuals (0.19–0.26%; n = 178) after the incident headache visit. The overall rates of malignant brain tumors were 0.33% (0.28–0.39%; n = 133) for the early neuroimaging group and 0.11% (0.08–0.15%; n = 45) for the referent group (*P* < .001). Table 3A summarizes the key malignant tumor diagnostic statistics for both groups.

**Table 3:** Cancer diagnosis statistics (total, primary, and metastatic) for the “Early neuroimaging” and “No early neuroimaging” groups: base definition, more restrictive, and less restrictive.

	Base definitions			More restrictive			Less restrictive		
	Early neuroimaging (n = 40,028)	No early neuroimaging (n = 40,028)	*P*-value	Early neuroimaging (n = 40,028)	No early neuroimaging (n = 40,028)	*P*-value	Early neuroimaging (n = 40,028)	No early neuroimaging (n = 40,028)	*P*-value
All malignant brain tumors, % (n)									
- Total	0.33 (133)	0.11 (45)	< .001	0.17 (69)	0.03 (13)	< .001	0.58 (234)	0.20 (80)	< .001
- Primary	0.20 (80)	0.06 (25)	< .001	0.14 (57)	0.03 (11)	< .001	0.41 (164)	0.12 (49)	< .001
- Metastatic	0.13 (53)	0.05 (20)	< .001	0.03 (12)	<0.01 (2)	0.008	0.17 (70)	0.08 (31)	< .001
Malignant brain tumors, <1-year post-headache, % (n)									
- Total	0.28 (114)	0.04 (15)	< .001	0.16 (65)	0.01 (3)	< .001	0.49 (194)	0.08 (32)	< .001
- Primary	0.19 (77)	0.02 (7)	< .001	0.14 (57)	0.01 (3)	< .001	0.38 (151)	0.05 (19)	< .001
- Metastatic	0.09 (37)	0.02 (8)	< .001	0.02 (8)	0.00 (0)	0.005	0.11 (43)	0.03 (13)	< .001
Median time to malignancy diagnosis: <1-year post-headache, days									
- Total	8	72	< .001	6	66	0.007	12	112.5	< .001
- Primary	6	66	< .001	5	66	0.004	10	85	< .001
- Metastatic	26	171.5	< .001	12.5	—	—	17	189	< .001
Malignancy diagnosis rate: 1+ years post-headache, number per 100,000 person-years									
- Total	11.7	18.3	0.12	2.5	6.1	0.12	24.6	29.3	0.41
- Primary	1.8	11.0	0.001	—	4.9	0.004	8.0	18.3	0.01
- Metastatic	9.8	7.3	0.45	2.5	1.2	0.45	16.6	11.0	0.18

**Fig 1:**
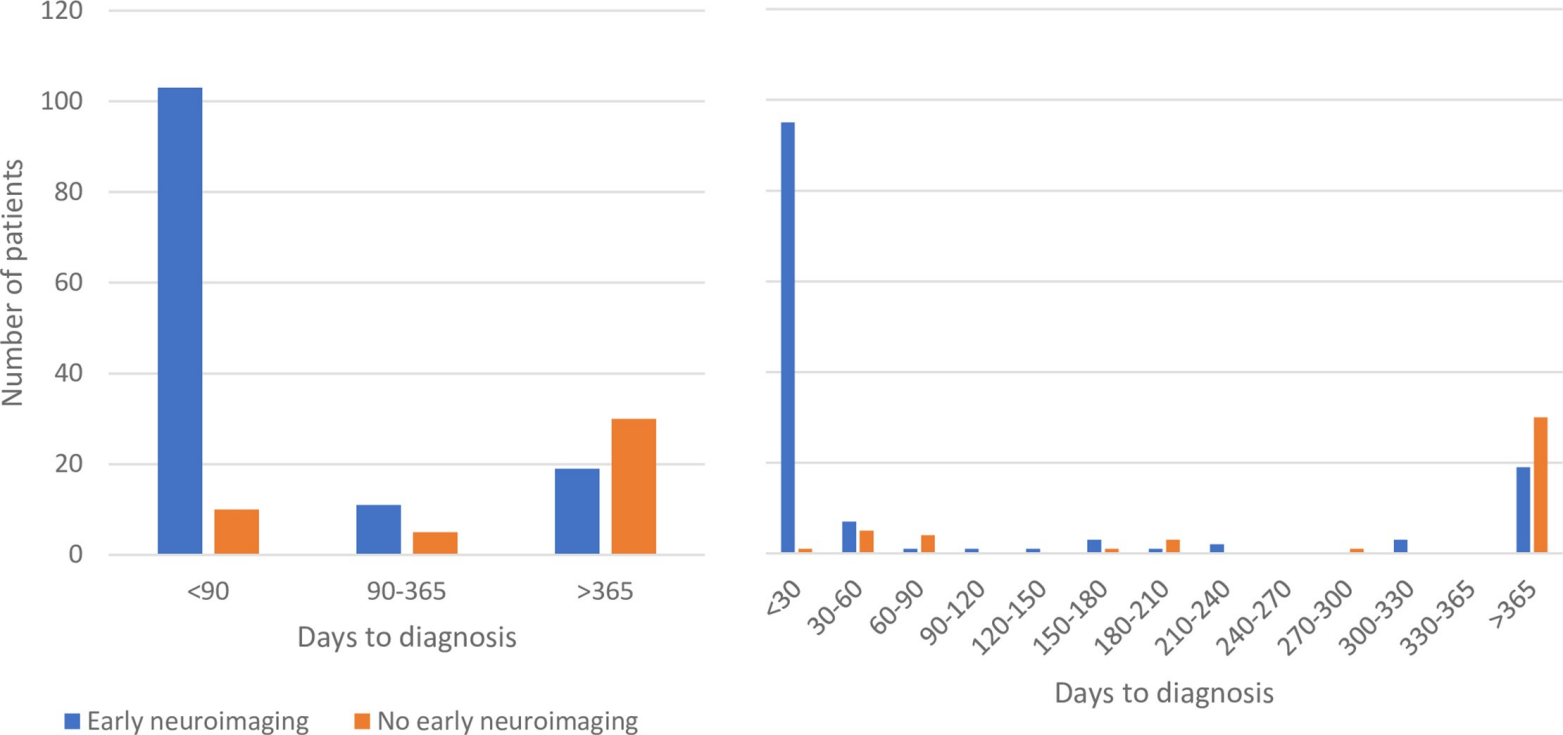
Histogram of individuals with cancer diagnoses from the imaged group and their matched controls by number of days from incident headache to cancer diagnosis.

In the first year after the incident headache visit, malignant brain tumors had been diagnosed at both higher rates and more quickly in the early neuroimaging group. Malignancies were diagnosed in 0.28% (0.23–0.34%; n = 114) of the early neuroimaging group and 0.04% (0.02–0.06%; n = 15) of the referent group in the first year (*P* < .001). The median time to cancer diagnosis in the first year following the incident headache visit was 8 (3–19) days in the early neuroimaging group and 72 (39–189) days in the referent group (*P* < .001). The largest differences in diagnosis of malignant brain tumors are within 90 days of diagnosis (Fig 1), after which the rates converge.

More than one year after the incident headache visit, the rate of malignant tumor diagnosis did not differ significantly between the early neuroimaging and referent groups: 11.7 (7.0–18.2) and 18.3 (12.4–26.1) per 100,000 person-years, respectively (*P* = .12).

More primary brain tumors were diagnosed relative to brain metastases: 0.13% (0.11–0.16%; n = 105) and 0.09% (0.07–0.11%; n = 73) of individuals, respectively (Table 3). In both groups, malignancies were diagnosed more quickly in the early neuroimaging group versus the referent group (*P* < .001). The frequency of diagnosis of all malignant brain tumors also converged in both groups after the first year post-incident headache, though fewer primary malignancies were diagnosed in the early neuroimaging group relative to the referent group after 1 year: 1.8 versus 11.0 per 100,000 person-years, respectively (*P* = 0.001). Table 3 provides the same results using the different tumor definitions. Results were similar to those found using the base definitions, though with fewer diagnoses under the more-restrictive definitions and more diagnoses under the less-restrictive definitions.

### Overall survival

**Fig 2:**
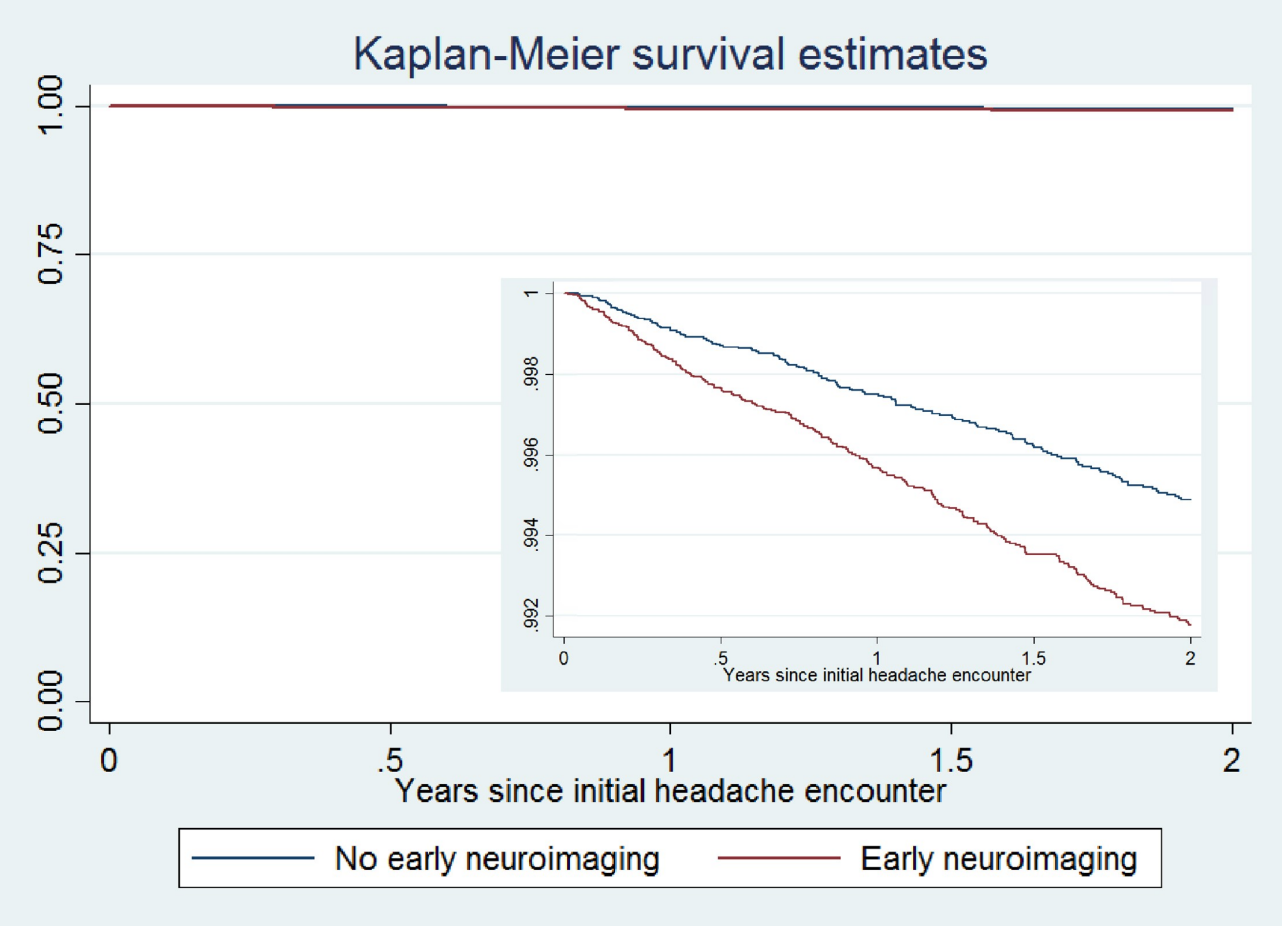
Kaplan-Meier survival curve for the early neuroimaging and non-early neuroimaging groups. Survival is measured from the incident headache date, in years.

Fig 2 shows a Kaplan-Meier survival curve for early neuroimaging and referent groups for the two years post-headache diagnosis. The risk of death was higher in the early neuroimaging group compared to the referent group, suggesting that any treatment-related benefits that accrued from early neuroimaging were not great enough to offset the likely higher disease severity in this group (hazard ratio = 1.64 [1.34–2.00], *P* < .001).

### Likely incidental findings

In the year following the incident headache, likely incidental findings were discovered in 3.17% (3.00–3.34%; n = 1,267) of individuals who received early neuroimaging and 0.66% (0.58–0.74%; n = 263) in the referent group (*P* < .001). In the early neuroimaging group, 83.5% (n = 1,058) of these likely incidental findings were diagnosed within 90 days of the incident headache. After 365 days, likely incidental findings were discovered at an annual rate of 261.3 (239.5–284.5; n = 530) per 100,000 person-years in the early neuroimaging group and 255.8 (234.3–278.8; n = 519) in the referent group (*P* = .73).

## Discussion

In this national cohort study exploring early headache neuroimaging, we found that the rate of malignant brain tumors among patients presenting for the first time with headaches is low: 303 such individuals must be imaged to diagnose a single malignancy. Furthermore, our data suggest that current headache neuroimaging practices rarely result in missed or delayed malignant brain tumor diagnoses. Finally, neuroimaging is much more likely to identify probable incidental findings than malignancies. Providers should consider these data in the context of the full clinical picture before referring patients for neuroimaging.

Providers naturally worry about missing the worst possible diagnosis—in the case of headache, a malignant brain tumor. A missed malignant brain tumor diagnosis is particularly worrisome because while not curable, treatment leads to an increase in survival [16, 27, 28]. Retrospective case series suggest that failing to image individuals with isolated headaches may lead to considerable rates of missed glioma diagnoses as 11% of these individuals putatively present with an isolated headache phenotype [29]. Our data quantify such a concern and demonstrate that few malignant brain tumors are missed. More than 1 year after the incident headaches, the annual frequency of malignancies was similar between those who received early neuroimaging and those who did not, suggesting that most of the late malignancy diagnoses were not “missed” diagnoses but were more likely to have been unrelated to the initial headache or undetectable at that time. If that were not the case, one would expect the frequency of diagnosis to be higher in the referent group after 1 year to capture both “missed” and new diagnoses.

While early neuroimaging rarely identifies malignant brain tumors, it likely has both modest benefits and modest harms in some patients. A likely benefit of early neuroimaging individuals with headaches is that it results in more rapid malignant brain tumor diagnosis. When looking only at the one-year window post-headache, malignant tumors are diagnosed 64 days earlier in individuals that receive early neuroimaging compared to those that do not. At the same time, while the yield of headache neuroimaging for malignancies is not inconsequential, it is still low—for every 1,000 headache individuals that receive neuroimaging studies, approximately 3.3 would eventually have a malignant tumor diagnosed, of which 2.6 would be identified in the first 90 days after the study and 0.5 would be identified more than one year later. Its plausible that early detection may have a survival benefit, but whether this is the case and the magnitude of any benefit is not well quantified. The most likely harm of early neuroimaging is the identification of incidental findings: for every 1,000 headache individuals imaged, 31.7 would receive such a diagnosis, consistent with the findings of others [6–8, 30]. Such incidental findings have the potential to lead to further testing, cost, patient risks, and patient anxiety.

### Limitations

Our study had several limitations. First, as a retrospective cohort analysis, our early neuroimaging and referent groups, even after matching, almost certainly vary on important outcome predictors such as headache time course and severity. Second, it is difficult to definitively determine whether the neuroimaging studies were truly “for headache” or merely performed in individuals “with headache”. Third, our analysis focuses on malignancies—benign tumors may also have clinical relevance, though they are generally less worrisome than gliomas or metastases. Fourth, using administrative data, we could not identify clinically relevant “red flags”, such as an abnormal neurologic examination or other associated symptoms, that would warrant appropriate neuroimaging.

### Conclusions

The decision about which individuals should receive neuroimaging for headaches requires weighing the potential benefits and risks. Our findings can help inform this decision. The rate of cancer diagnosis in our study likely represents a ceiling frequency of malignant tumors in individuals with headaches. Given the lack of clinical detail in our dataset, it is likely that many of the individuals that received early neuroimaging had “red flags” (e.g., focal neurologic signs) that would have led to clearer decisions to obtain neuroimaging. Conversely, our rate of incidental findings likely underestimates the true rate of incidental findings as our definition was relatively limited and many incidental findings likely were not registered in visit diagnoses. Conceptually, it may be more helpful to think about the decision to refer for neuroimaging as centered on other focal, neurological findings rather than simply the fact that the individuals have headaches.

## Supporting information

S1 File Diagnostic and procedure codes for analyses. (PDF)

S1 Table Descriptive statistics for the “Early neuroimaging” and “No early neuroimaging” cohorts (not matched). (PDF)
