# Supplementary material for: Impact of early headache neuroimaging on time to malignant brain tumor diagnosis: A retrospective cohort study
Source: PLoS One. 2019 Feb 1;14(2):e0211599. doi: 10.1371/journal.pone.0211599 (PMC6358089; doi:10.1371/journal.pone.0211599)
Supplement: S1 File — (PDF) [file pone.0211599.s001.pdf]

S1 File. Diagnostic and procedure codes for analyses.

Codes for imaging: CPT codes 70450-70479, 70551-70553; ICD-9-CM procedure codes 807.3x, 889.1x

Codes for primary malignant brain tumor diagnosis: ICD-9-CM diagnosis codes 191.xx and 192.xx

Codes for surgical intervention: CPT codes 00211, 0128x, 61304, 61510, 61518, 61796, 61797, 61799, 63620, and 63621

Codes for radiation therapy: CPT codes 77261-77263, 77280, 77285, 77290, 77295, 77334, 77401-77416, 77418, 77432, and 77435

Code for brain metastasis diagnoses: ICD-9-CM diagnosis code 198.3

Code for chemotherapy (i.e., anti-neoplastic agents): American Hospital Formulary Service (AHFS) class 10.00

Codes for likely incidental findings: ICD-9-CM diagnosis codes 212.0, 215.0, 224.5-9, 225.0-4, 225.8, 227.3-5, 237.0-1, 237.5, 239.6, 348.0, 377.51-2, and 377.71

Codes for smoking history: ICD-9-CM diagnosis codes 305.1 and V15.82

Codes for prior neurological symptoms: ICD-9-CM diagnosis codes 784.3, 784.4, 784.5, 780.1, 780.3, 780.4, 780.93, 780.97, 781.0, 781.1, 781.2, 781.3, 781.4, 781.8, 781.94, 782.0, 728.97

Codes for prior cancer diagnoses: CCS categories 11-44
