# Supplementary material for: Impact of early headache neuroimaging on time to malignant brain tumor diagnosis: A retrospective cohort study
Source: PLoS One. 2019 Feb 1;14(2):e0211599. doi: 10.1371/journal.pone.0211599 (PMC6358089; doi:10.1371/journal.pone.0211599)
Supplement: S1 Table — (PDF) [file pone.0211599.s002.pdf]

S1 Table. Descriptive statistics for the "Early neuroimaging" and "No early neuroimaging" cohorts (not matched).

	Early neuroimaging (n=40,028)	No early neuroimaging (n=140,595)	Standardized difference
Female, %	59.94	66.19	-0.130
**Enrollment duration categories**			0.018
<6 years, %	34.12	35.91	
6-8 years, %	27.19	26.88	
8+ years, %	38.69	37.21	
**Age categories**			0.225
18-34, %	29.51	35.14	
35-49, %	38.79	40.73	
50-64, %	19.99	18.69	
65+, %	11.71	5.44	
Migraine diagnosis, %	15.15	29.67	-0.354
Prior neurological symptoms, %	12.58	8.40	0.137
Smoking history, %	5.92	5.31	0.026
**Charlson comorbidities**			
Myocardial infarction, %	0.58	0.29	0.044
Congestive heart failure, %	0.96	0.46	0.060
Peripheral vascular disease, %	0.02	0.02	0.003
Cerebrovascular disease, %	0.02	0.02	0.001
Dementia, %	<0.01	<0.01	0.001
Chronic pulmonary disease, %	15.38	14.72	0.018
Rheumatic disease, %	1.57	1.24	0.028
Peptic ulcer disease, %	1.18	0.98	0.003
Mild liver disease, %	0.35	0.22	0.021
Diabetes without chronic complication, %	7.70	5.41	0.093
Diabetes with chronic complication, %	1.25	0.60	0.067
Hemiplegia or paraplegia, %	0.04	0.02	0.008
Renal disease, %	1.48	0.72	0.073
Any malignancy, including lymphoma and leukemia, except malignant neoplasm of skin, %	0.02	0.05	-0.011
Moderate or severe liver disease, %	0.07	0.06	0.005
Metastatic sold tumor, %	--	<0.01	-0.004
AIDS/HIV, %	0.29	0.17	0.025
